# The Influence of Virus Infection on the Extracellular pH of the Host Cell Detected on Cell Membrane

**DOI:** 10.3389/fmicb.2016.01127

**Published:** 2016-08-17

**Authors:** Hengjun Liu, Hisataka Maruyama, Taisuke Masuda, Ayae Honda, Fumihito Arai

**Affiliations:** ^1^Department of Micro-Nano Systems Engineering, Nagoya UniversityNagoya, Japan; ^2^Department of Frontier Bioscience, Hosei UniversityTokyo, Japan

**Keywords:** influenza virus, FITC, pH sensor, extracellular pH, immunostaining

## Abstract

Influenza virus infection can result in changes in the cellular ion levels at 2–3 h post-infection. More H^+^ is produced by glycolysis, and the viral M2 proton channel also plays a role in the capture and release of H^+^ during both viral entry and egress. Then the cells might regulate the intracellular pH by increasing the export of H^+^ from the intracellular compartment. Increased H^+^ export could lead indirectly to increased extracellular acidity. To detect changes in extracellular pH of both virus-infected and uninfected cells, pH sensors were synthesized using polystyrene beads (ϕ1 μm) containing Rhodamine B and Fluorescein isothiocyanate (FITC). The fluorescence intensity of FITC can respond to both pH and temperature. So Rhodamine B was also introduced in the sensor for temperature compensation. Then the pH can be measured after temperature compensation. The sensor was adhered to cell membrane for extracellular pH measurement. The results showed that the multiplication of influenza virus in host cell decreased extracellular pH of the host cell by 0.5–0.6 in 4 h after the virus bound to the cell membrane, compared to that in uninfected cells. Immunostaining revealed the presence of viral PB1 protein in the nucleus of virus-bound cells that exhibited extracellular pH changes, but no PB1 protein are detected in virus-unbound cells where the extracellular pH remained constant.

## Introduction

The influenza virus can infect a wide range of vertebrate species, resulting in changes in the activity of host ATPase (Guinea and Carrasco, 1995) as well as in the cellular ion levels (Pinto et al., 1992). Several studies have reported that large amounts of RNA are synthesized within a short period after the influenza virus enters the cell, thereby suggesting that the rate of ATP consumption would be higher in influenza virus-infected cells than in uninfected cells (Guinea and Carrasco, 1995; Hui and Nayak, 2001). The virus-infected cells synthesize some ATP by oxidative metabolism, and some by glycolysis. Glycolysis is the metabolic pathway that converts glucose (C_6_H_12_O_6_) into pyruvate (CH_3_COCOO^−^ + H^+^). Pyruvate can be converted into lactate reversibly. High rates of glycolysis and lactate are reported as a common feature of virus-infected cells (Allison, 1963; Singh et al., 1974). This follows from the intimate association between lactate and H^+^ gradient across the cell plasma membrane (Allison, 1963). One obvious hypothesis is that the export of metabolic acids (lactate and CO_2_) and H^+^ from the cell into the near-surroundings will acidify the extracellular compartment. Some researchers have reported a reduction in intracellular pH (pH_i_) of virus-infected cells (Steinhauer et al., 1991; Ciampor et al., 1992). But no researches have reported the extracellular pH (pH_e_) change induced by virus infection. The investigation on pH_e_ can improve our understanding of the metabolic pathway of the cell and also the pH gradients inside and outside the cell membrane.

Measurements of pH in cells and other micro-environments have largely been carried out using responsive fluorescence dyes. Recently, pH responsive sensors based on fluorescence dyes have been developed and are used in imaging and measurements in living cells and small environments (Oyama et al., 2012; Yin et al., 2012). They have the advantage of stable fluorescence, require low stimulus levels for activation, and enable single cell measurement. Thus, these fluorescence pH sensors have the potential to be used in the pH measurements of virus-infected cells. In our previous work (Liu et al., 2014), pH sensors were synthesized using polystyrene beads (ϕ1 μm) containing Rhodamine B and Fluorescein isothiocyanate (FITC). The fluorescence intensity of FITC can respond to both pH and temperature. So Rhodamine B was also introduced in the sensor for temperature compensation. After temperature compensation, the pH can be measured by fluorescence changes of FITC. In this paper, the pH_e_ changes of the cell after influenza virus infection were investigated using the synthesized pH sensor by adhering the sensor to the cell membrane. The reasons for pH_e_ changes and the role of different ion channels will be also discussed in this paper.

## Materials and methods

### Cell culture

Madin-Darby canine kidney (MDCK) cells were used for experiments. Prior to injection, the cells were cultured in a glass-based dish (ϕ 3 cm, Asahi Glass Co. Ltd., Japan) and incubated at 37°C, bubbled with 95% air, and 5% CO_2_ gas. Eagle Minimum Essential Medium (EMEM) containing 10% fetal bovine serum (FBS) was used as the cell medium.

### Fluorescence labeling of virus

Influenza virus A/Puerto Rico/8/34 (H1N1) (wild type) was propagated in 10-day-old embryonated chicken eggs. The influenza virus (in allantoic fluid) was incubated with Syto21 (2 μg/mL in PBS) for 30 min at room temperature. The virus solution was centrifuged for 2 min at 700 × g using a spin column containing Sephadex G50 beads (Pharmacia, USA) to remove the excess dye. The flow-through virus solution was then used in further experiments.

### Preparation of the pH sensor

As described in our previous work (Liu et al., 2014), polystyrene microbeads (ϕ1 μm) with amino-group modified surfaces were used as the sensor carriers. FITC has been used in pH sensor (Liu et al., 2000), but our previous study showed that temperature could affect the pH sensitivity of FITC. In order to fabricate the pH sensor, FITC was modified on the bead surface for pH sensing, while Rhodamine B was introduced inside the bead for temperature compensation. First, a solution of amino-polystyrene beads and 1 g/L Rhodamine B (in alcohol) (1:1 v/v) was stirred for 5 min and then washed with deionized (DI) water. The beads were then added to an FITC saturated aqueous solution for 1 h, followed by three washes with DI water.

### Virus infection

MDCK cells were cultured with influenza viruses labeled with Syto 21 for 15 min at 34°C in serum-free medium (1000 copies/ml). After 15 min, some of the viruses were bound to the cell membranes, following which the serum-free medium containing unbound virus was replaced with a new serum-containing medium.

### pH_e_ measurement of influenza virus-bound and unbound cells

**Figure 1:**
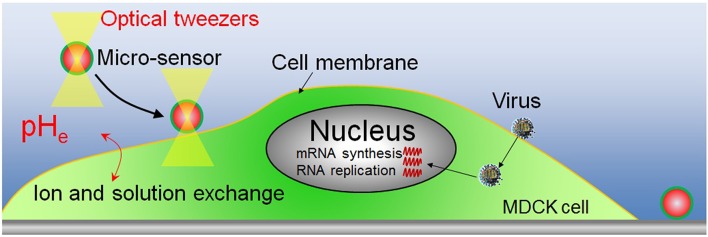
**Schematic of the pH_**e**_ measurement of virus-infected cell by pH sensor adhered on cell membrane**.

After removing the unbound virus particles, the complete medium and pH sensors were added to the dish. The pH sensor was then transferred and attached to the surface of virus-bound and virus-unbound cells by using optical tweezers. A near-infrared laser, considered to be safe for cells, was employed for the optical tweezers (Maruyama et al., 2009). The maximum power of the laser was over 5 W and its wavelength was 1064 nm. The optical tweezers have been used widely in the transfer of small objects (nm-μm), such as micro-sensor (Maruyama et al., 2013) and a single virus (Masuda et al., 2014). Figure 1 shows a schematic of the pH_e_ measurement of virus-infected cell by pH sensor adhered on cell membrane. The sensor is adhered on cell membrane and it is also placed in the extracellular environment. So the pH sensor which adhered to cell membrane can detect the pH_e_ changes close to the cell membrane. pH_e_ changes are closely related with the ions and solutions exchanges between the both sides of cell membrane. The other micro-sensor is adhered on the substrate as the contrast sensor. By detecting the fluorescence changes of the sensor adhered on substrate, the fluorescence changes of FITC induced by the excitation light can be investigated. Then fluorescence changes of the sensor (on cell membrane) induced by virus infection can be investigated with the compensation of fluorescence fading resulted from excitation light. The fluorescence intensity of FITC of the sensors was measured for about 6 h after the sensor was adhered to the cell surface using a Nikon TiE microscope fitted with a 100 × objective lens. All experiments were carried out in the culture chamber (5% CO_2_, 34°C).

### Experimental system setup

The fluorescence image of the target was obtained from an inverted confocal microscope (Ti-E Nikon) equipped with a high magnification lens (Plan Fluor 100 ×, Nikon) and CCD camera (iXon, Andoe). Wavelengths of 470 and 532 nm were selected as the excitation wavelengths for FITC and Rhodamine B, respectively. Rhodamine B is excited by green light (532 nm) and emits red fluorescence (peak wavelength 585 nm). FITC is excited by blue light (470 nm) and emits green fluorescence (peak wavelength 520 nm). The fluorescent images can be taken automatically in a certain time interval (30 min in our experiment).

### Immunostaining of the cells

After the measurement experiments, all cell in the glass-based dish was immunostained with anti-PB1 (after 6 h post-infection). Actually, we marked the cells which have been observed for pH sensing in Section pH_e_ Measurement of Influenza Virus-bound and Unbound Cells and then found the same cells in the glass-based dish after immunostaining. The cells were washed three times with phosphate-buffered saline (PBS) and fixed with 4% paraformaldehyde solution at room temperature for 10 min. They were re-washed three times with PBS, treated with 0.5% Triton X-100 for 5 min, and blocked with 1% bovine serum albumin (BSA) in PBS at room temperature. Lastly, the cells were incubated with anti-PB1 antiserum for 1 h at 37°C and washed by 1% BSA/PBS solution, following which the cells were incubated with anti-rabbit IgG conjugated with Alexa 488 for 1 h at 37°C. The cells were observed under a microscope fitted with a 100 × objective lens (Zeiss LSM 510 META).

## Results

### Virus infection and pH sensor adhesion

**Figure 2:**
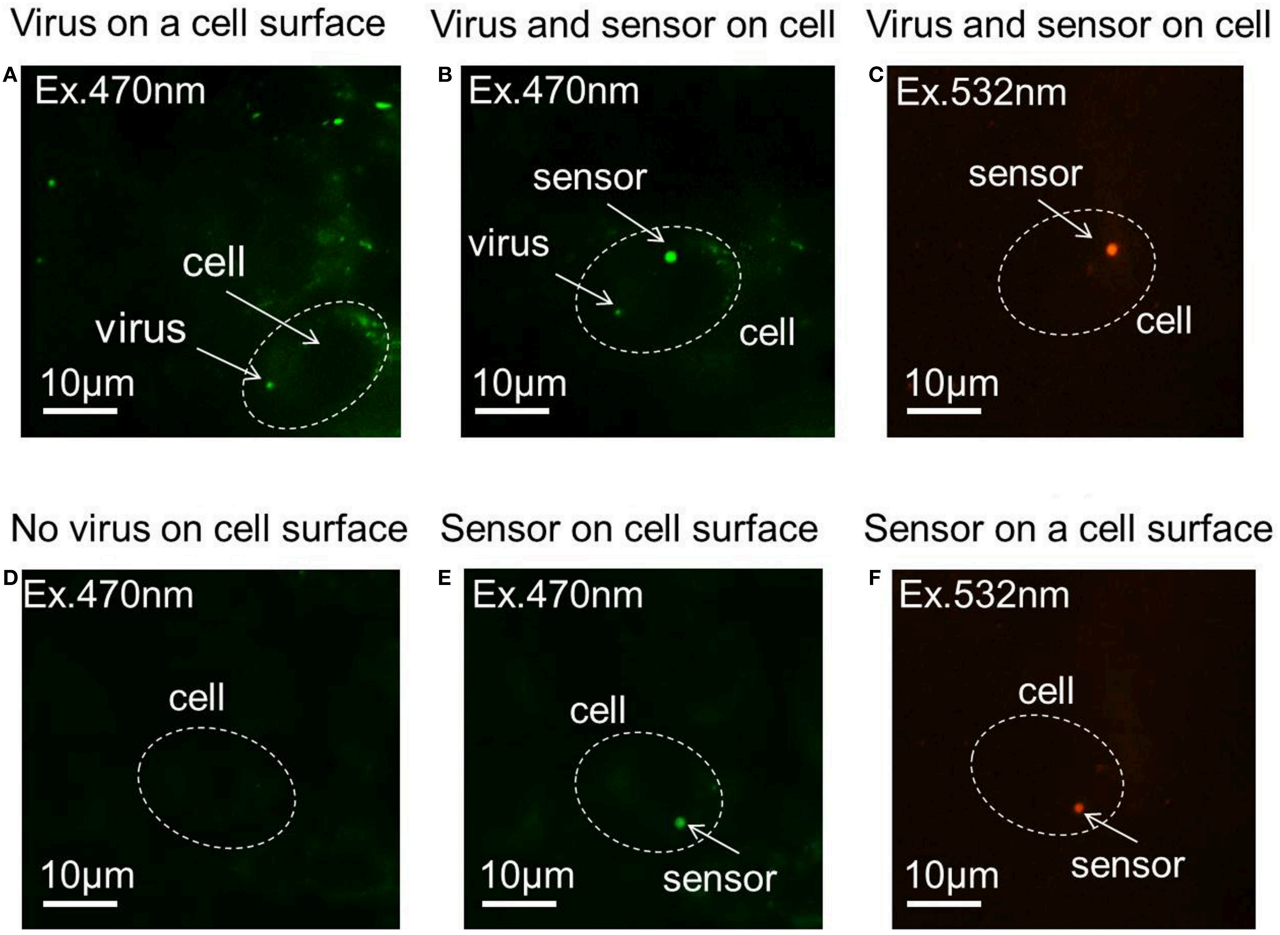
**Fluorescence images of the pH sensor and virus on the cell membrane. (A)** Virus adhered to the cell surface. **(B)** A pH sensor adhered to the same cell with a virus on its surface, with an excitation of 470 nm laser. **(C)** A pH sensor adhered to the same cell with a virus on its surface, with an excitation of 532 nm laser. **(D)** Virus-unbound cell. **(E)** A pH sensor adhered to a virus-unbound cell, with an excitation of 470 nm laser. **(F)** A pH sensor adhered to a virus-unbound cell, with an excitation of 532 nm laser.

Figures 2A,D show virus-bound and virus- unbound cells, respectively. The virus is detected by the fluorescence of Syto 21. The pH sensor was then attached to virus-bound and virus–unbound cells using optical tweezers (Figures 2B,E), and the fluorescence of Rhodamine B and FITC can be observed under excitation sources of 532 and 470 nm, respectively. The sensor and virus that adhered to the surface of the same cell can be distinguished not only by their different sizes but also by the presence of fluorescence at 532 nm, as Syto 21 of the virus cannot be excited at that wavelength, but Rhodamine B of the sensor will fluoresce. Figures 2C,F show the fluorescence of Rhodamine B of the sensor, with no fluorescence observed from Syto 21 of the virus.

### pH changes in virus-bound and –unbound cells

**Figure 3:**
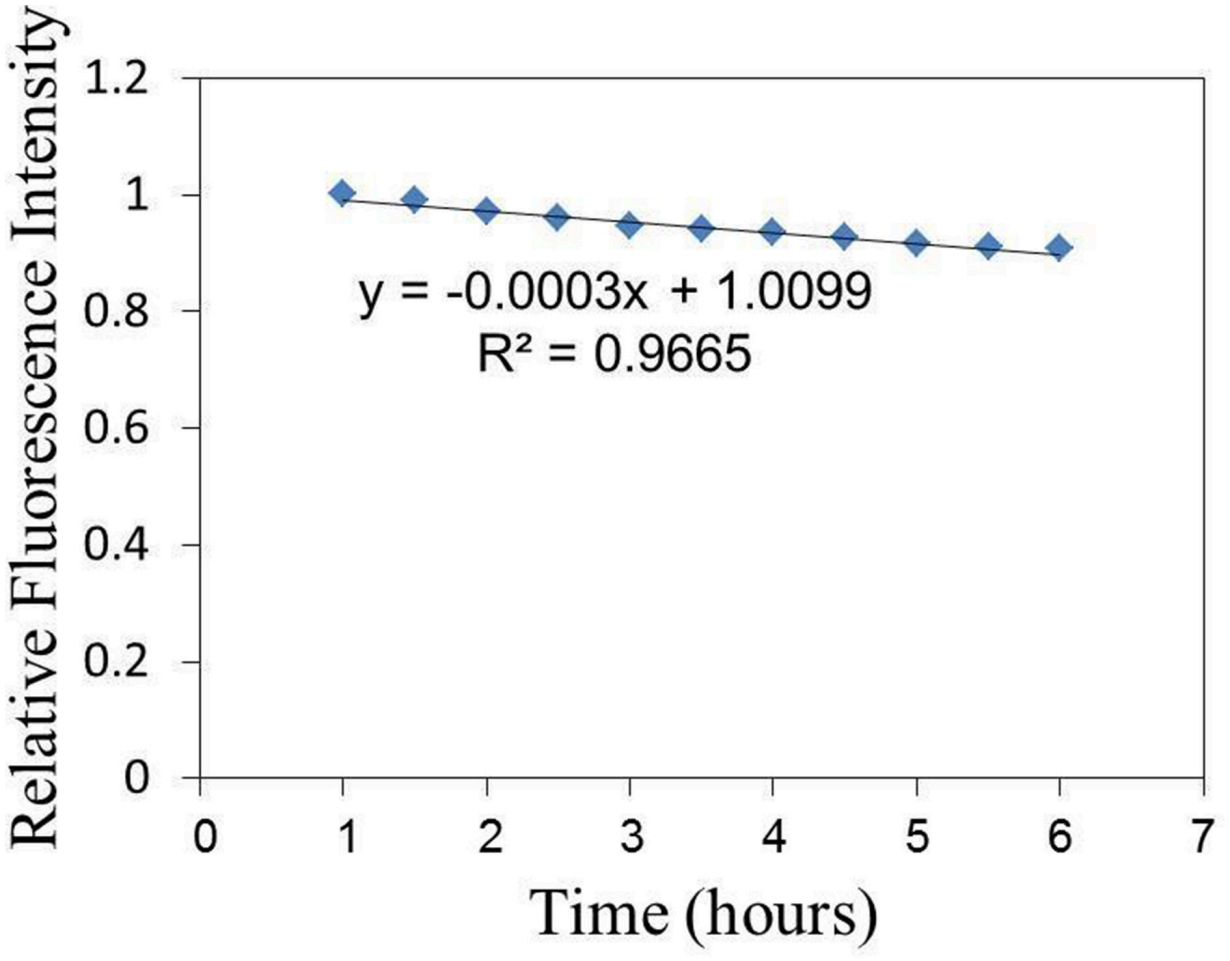
**The relative fluorescence intensity changes of FITC of the sensor adhered to substrate induced by the excitation light**.

**Figure 4:**
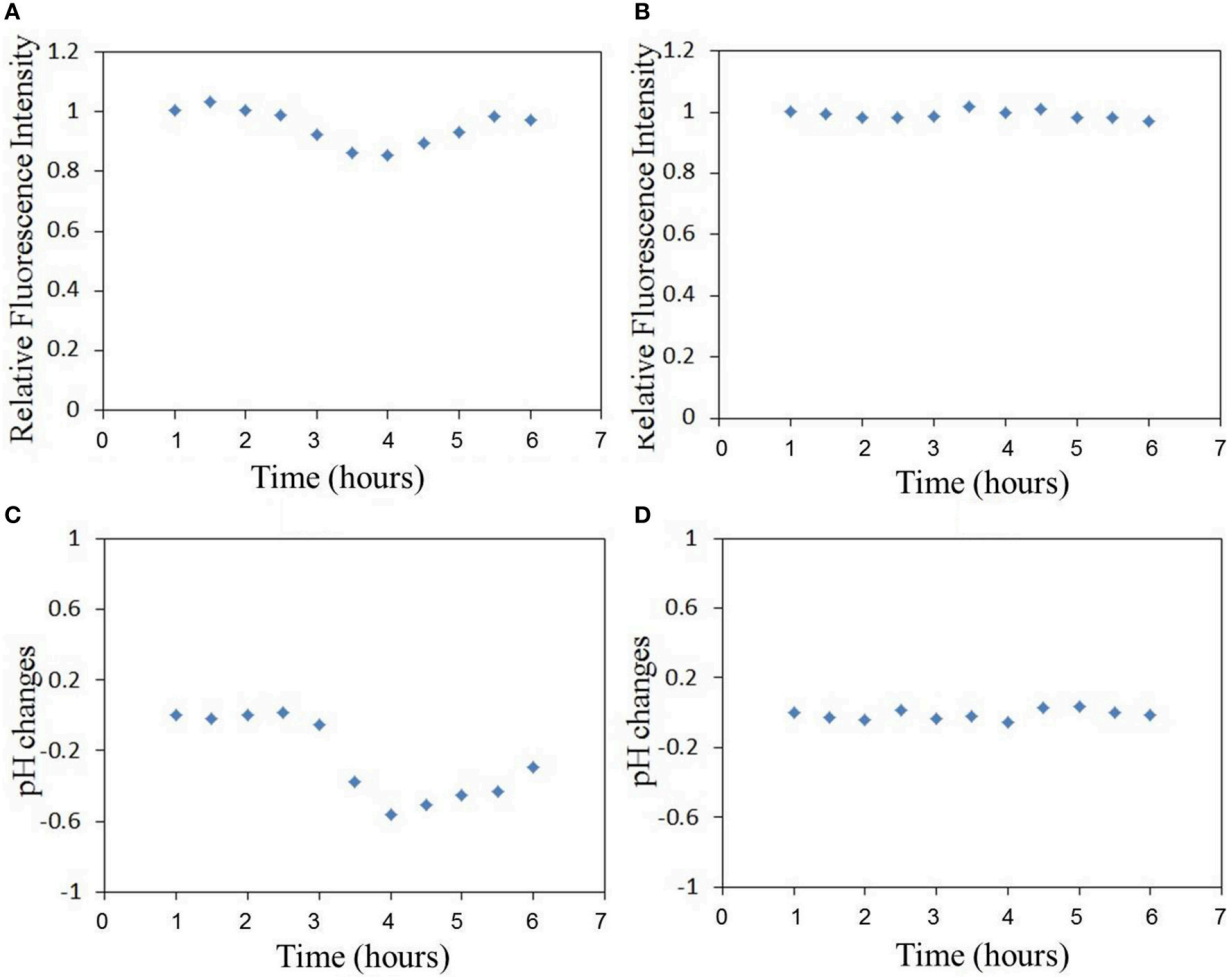
**The relative fluorescence intensity changes of FITC of the sensor adhered to (A) virus-bound cell and (B) virus-unbound cell, and the pH_**e**_ changes of (C) virus-bound cell and (D) virus-unbound cell calculated basing on the fluorescence changes in (A,B), (Time-course measurements are initiated post-virus binding to the cell membrane)**.

The relative fluorescence intensity changes of FITC of the sensor adhered to substrate induced by the excitation light are shown in Figure 3. The fluorescence intensity is decreased by the excitation light and decreasing with the excitation time. The relative fluorescence changes of the sensor (on cell membrane) induced by virus infection can be investigated with the compensation of fluorescence fading as shown in Figure 3. Figure 4 shows the relative fluorescence intensity changes of the sensors adhered to different cells in 6 h. Figure 4A shows the fluorescence intensity of the sensor which was adhered to the virus-bound cell decreased from 2 h after the virus adhesion. However, there was no obvious change in the fluorescence intensity of the sensor adhered to the virus-unbound cell as shown in Figure 4B.

Based on the sensitivity of FITC to pH changes as reported in our previous study (Liu et al., 2014), the pH_e_ changes can be calculated directly from the fluorescence intensity changes of FITC in Figure 4. It is important to note that there will be changes in cellular temperature after infection with the virus. Several studies have reported that large amounts of RNA are synthesized within a short period after the influenza virus enters the cell, thereby suggesting that the rate of ATP consumption would be higher in influenza virus-infected cells than in uninfected cells (Guinea and Carrasco, 1995; Hui and Nayak, 2001). A higher rate of ATP consumption compared to its metabolism in the cell will likely increase the temperature of the virus-bound cell. This temperature change can also be responsible for changing the FITC fluorescence, and thus the fluorescence intensity changes of FITC in Figure 4 could include two parts: those induced by pH changes and those by temperature changes. Then temperature compensation is necessary for pH calculation. In our previous work (Liu et al., 2014), the calibration and compensation of the pH sensor has been discussed. The fluorescence responses of FITC to pH at different temperatures have been detected. So the pH sensitivity of the sensor which is related to temperature has been summarized. If we can get the temperature information, the pH sensitivity of the sensor can be calculated. Then the pH change can be calculated basing on the pH sensitivity of the sensor and also the fluorescence intensity changes of the sensor. This is the method of temperature compensation which is aimed at removing the effect of temperature on pH measurement.

**Figure 5:**
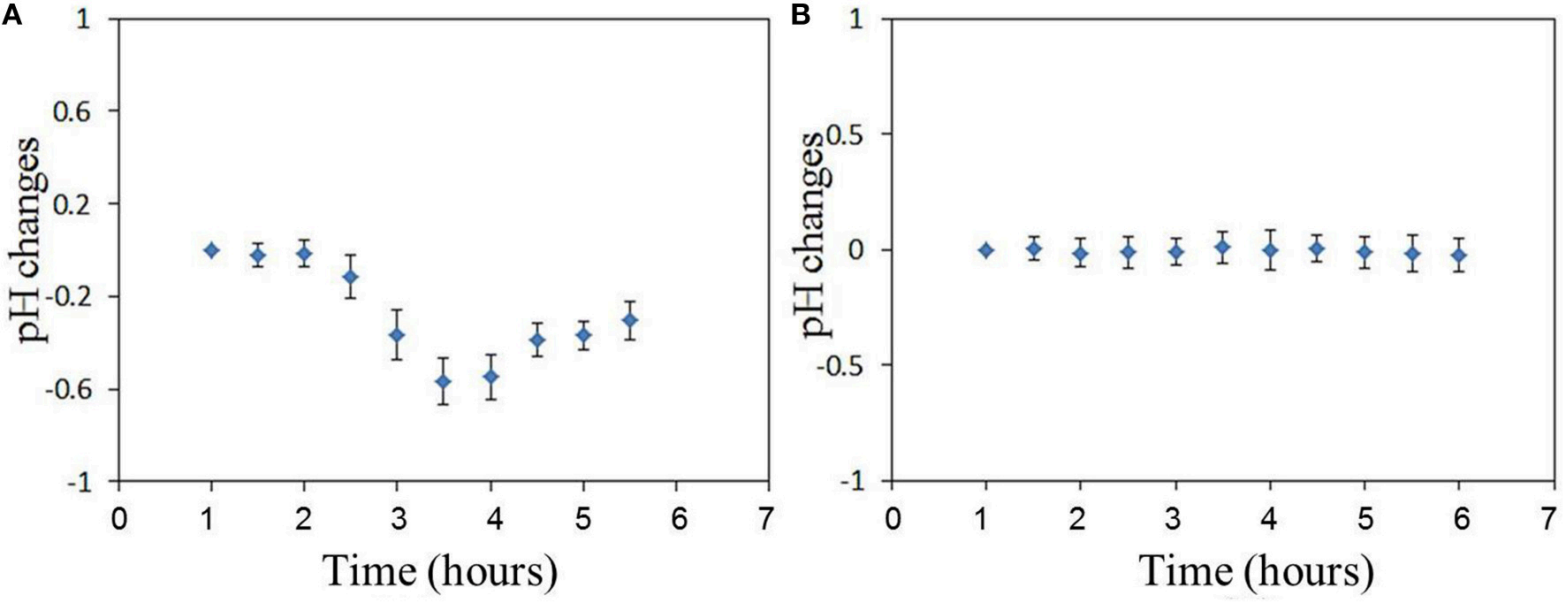
**The average pH_**e**_ changes of (A) virus-bound cells and (B) virus–unbound cells (***n*** = 8)**.

The results of pH_e_ changes of the virus-bound cell after temperature compensation are shown in Figures 4C,D. pH_e_ of influenza virus-bound cell decreased by approximately 0.55 in 4 h after virus binding. There was no obvious decrease in pH_e_ of uninfected cell (Figure 4D). The average pH_e_ changes of 8 virus-bound and 8 -unbound cells (from 3 different dish samples) are shown in Figure 5. The results show that pH_e_ of virus-bound cells decreases by 0.5–0.6 in 4 h after virus binding (Figure 5A). No obvious changes in pH_e_ were detected on virus-unbound cells (Figure 5B).

### Immunostaining of the cells

**Figure 6:**
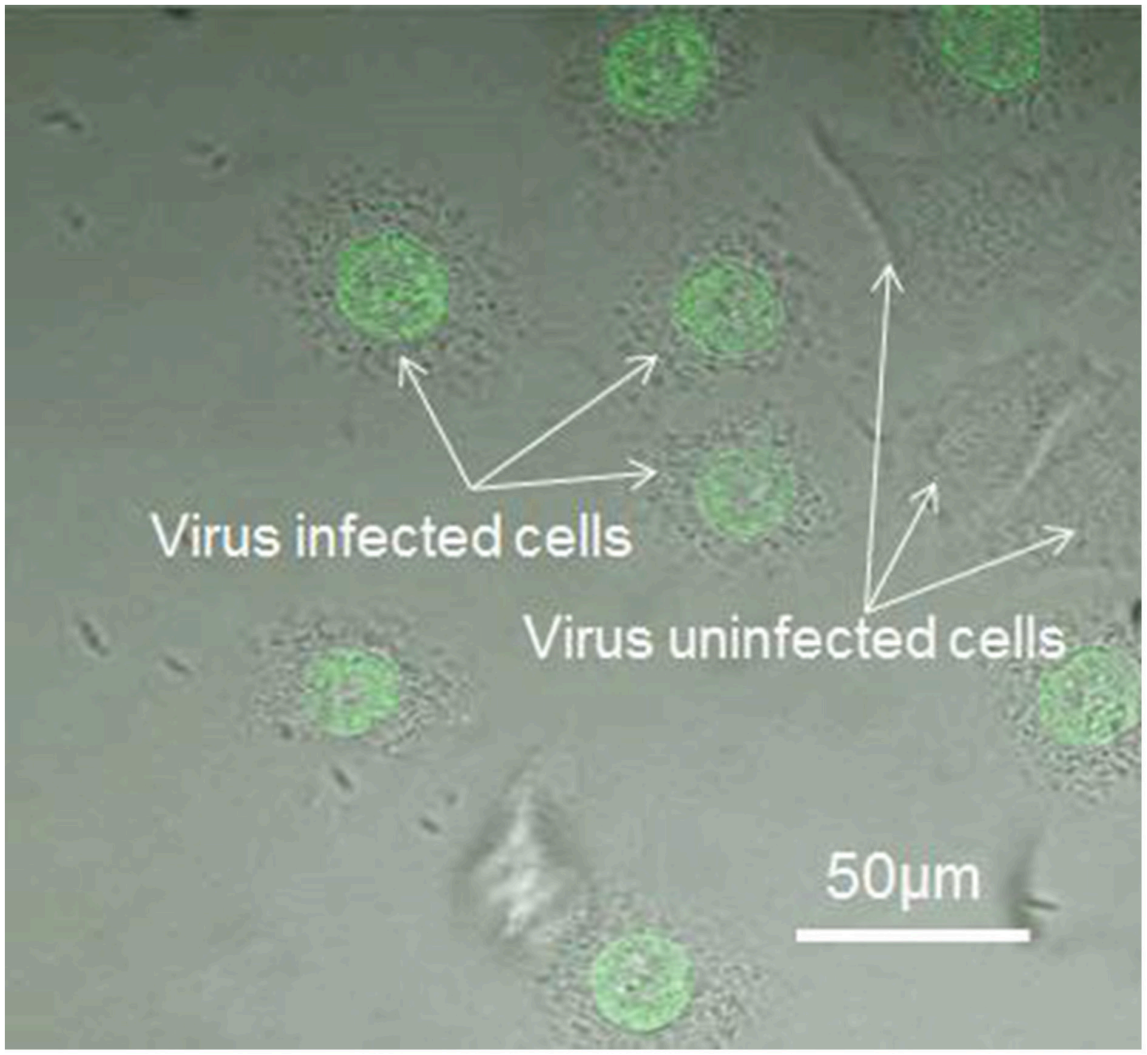
**Immunostaining of the virus-bound and -unbound cells (detected using anti-PB1 serum and an anti-rabbit IgG labeled with Alexa 488)**.

To confirm the viral infection after binding on the cell surface as well as viral replication in the cells, the cells (utilized in pH sensing in Section pH changes in virus-bound and –unbound cells) were immunostained with anti-PB1 antiserum after pH measurement. Since PB1 protein is known to be a part of the RNP complex involved with aiding viral genome replication, detection of PB1 protein within the nucleus of an infected cell is expected. We marked the cells which have been utilized in pH sensing and then found the same cells in the glass-based dish after immunostaining. Using this method, we can confirm the observed cells utilized in Figure 5A are infected successfully and the cells utilized in Figure 5B are not infected by virus. Figure 6 shows viral PB1 protein are detected in the nucleus of virus-bound cells (in one dish sample) that exhibited pH_e_ changes, but no PB1 protein are detected in virus-unbound cells where the pH_e_ remained constant. The virus-bound cells from other dish samples which are utilized in pH sensing have showed the same results. These results suggest that the virus replicates in the nucleus of the host cell induces pH_e_ changes. Our data clearly demonstrates a difference in pH_e_ near cell membrane between the influenza virus-infected and uninfected cells.

## Discussion

### Primary highlights of the study

In this study, we prepared a sensor based on Rhodamine B and FITC fluorescence, and successfully implemented it in the measurement of pH_e_ changes close to the cell membrane of influenza virus-infected and uninfected cells. We found that influenza virus multiplication decreased pH_e_ close to the cell membrane by approximately 0.5–0.6 units. Immunostaining revealed the presence of PB1 protein in the nucleus of virus-bound cells that exhibited pH_e_ changes, but not in virus-unbound cells where the pH_e_ remained constant. These results suggest that the influenza virus infection and proliferation in the host cell could induce a pH_e_ decrease near cell membrane.

### Proposed mechanisms of decrease in pH_e_ after virus infection

**Figure 7:**
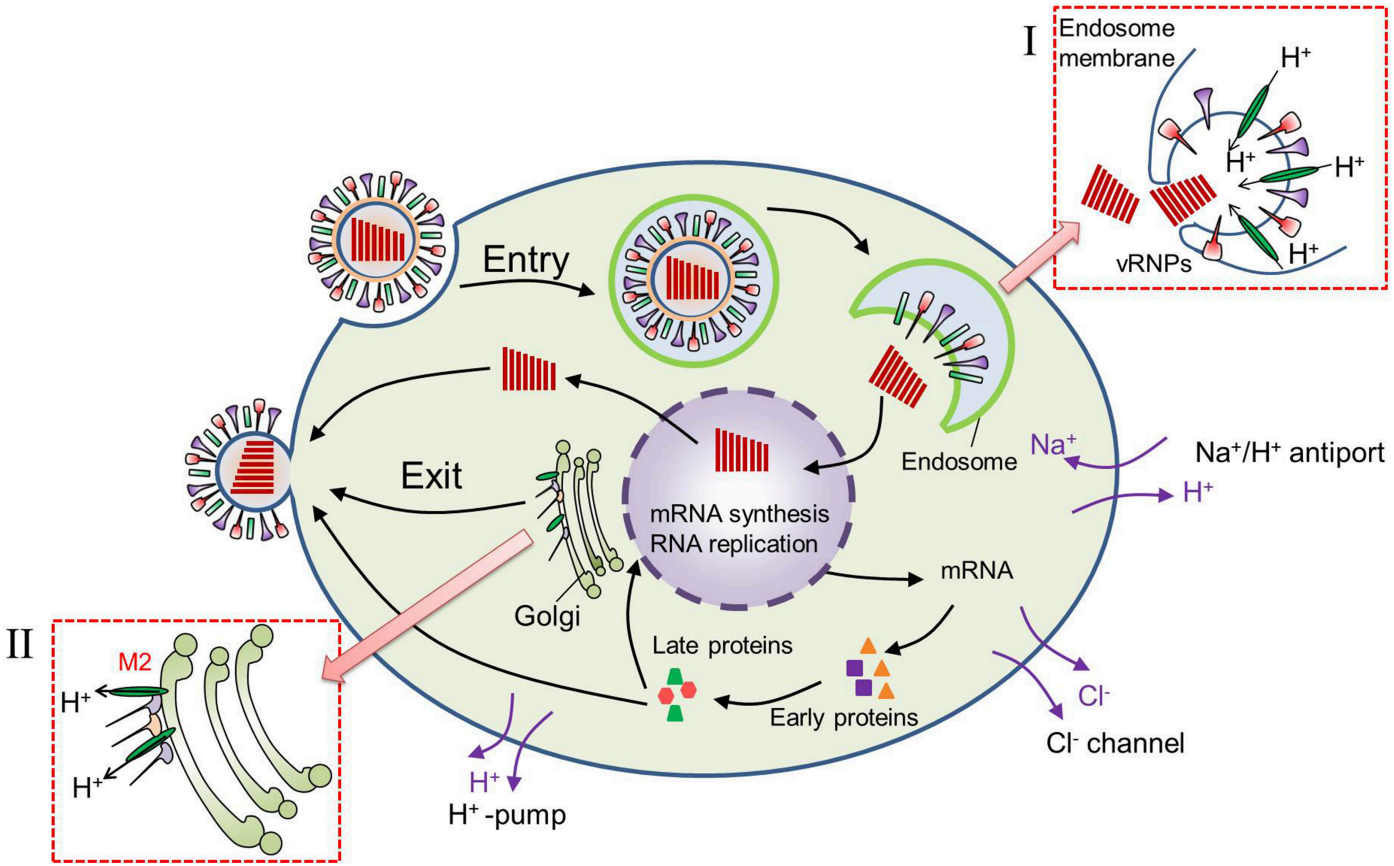
**Virus replication cycle after virus infection into a cell**. (I) During virus entry, M2 proton channel shunts H+ from the acidic endosome to the virion interior, leading to membrane fusion and release of the genome. (II) During virus exit, M2 shunts H+ from trans-Golgi network to the cytoplasm, preventing premature conformational changes in the viral hemagglutinin (HA).

The decrease in pH_e_ near the cell membrane after virus infection should be related with two factors: the H^+^ produced in the cytoplasm and its release into the extracellular environment. First, high rates of glycolysis are required to produce more ATP which is necessary for large amounts of virus replications in host cell. The glycolysis will produce more metabolic acids and H^+^ in cytoplasm. Actually, many researches have reported that there was 0.3–0.4 unit reduction in pH_i_ of virus-infected cells (Steinhauer et al., 1991; Ciampor et al., 1992). The decrease in pH_i_ of virus-infected cell is not only related with glycolysis but also the functions of M2 protein embedded in the viral lipid membrane. As shown in Figure 7, the well characterized M2 viroporin of influenza virus plays roles both during viral entry and egress. During entry, the M2 proton channel shunts H^+^ from the acidic endosome to the virion interior, leading to membrane fusion and then release of the genome as well as H^+^ initiation. The release of H^+^ to the cytoplasm results in a decrease in cytoplasmic pH. In certain subtypes, M2 also equilibrates the intraluminal pH of the trans-Golgi network with the cytoplasm, preventing premature conformational changes in the viral hemagglutinin (HA) during exit (Takeda et al., 2002; Pinto and Lamb, 2006; Betakova, 2007). This results in pH increase inside the trans-Golgi network and a pH decrease in cytoplasm.

Then the cells might regulate the pH_i_ by increasing the export of H^+^ from the intracellular compartment (Gillies et al., 1992). Export of increased H^+^ could lead indirectly to increased extracellular acidity. As we know that there are many ion channels on cell membrane as shown in Figure 7. Cl^−^ channels are known to take part in the transfer of water and ions, and volume regulating of cells by modulating a volume regulated anion current (Sardini et al., 2003). Na^+^/ H^+^ exchange and H^+^–pump in the plasma membrane are reported to be related with the control and regulation of pH_i_ (Harvey, 1988). So the pH_i_ can be regulated by H^+^ export from cell to maintain its normal pH, and the pH_e_ will decrease as a result of H^+^ export.

### Potentials in local pH_e_ measurement of a single cell

Many researches were focused on the investigation of pH_i_ after virus infection. For example, Moore et al. (1988) reported that sindbis virus infection decreased the intracellular pH by approximately 0.5 as measured using a pH-sensitive fluorescence probe. But the measurement of pH_e_ as well as the pH gradients on both sides of cell membrane has not been investigated very well. Since individual cells can differ dramatically in size, protein levels, and expressed RNA transcripts. Single cell analyses are needed to better understand cellular responses in tissues and complex environments, and would give an accurate assessment of the behavior of the cell as one cell is studied at a time. Our proposed pH sensor which is fabricated from polystyrene microbeads can be used on the investigation of pH_e_ for a single cell near the cell membrane. It can be adhered to a special part of the cell, allowing local pH_e_ measurements of a single cell.

### Future research

The data from the surface virus-bound cells clearly demonstrated that there was a difference in pH_e_ between the influenza virus-infected and uninfected cells. On the basis of our discovery, we can also examine the pH of other virus-infected cells. We consider that pH changes in virus-infected cells may differ among viruses. Thus, further studies will be required to understand the virus specificity amounting to different pH changes. Secondly, the pH_i_ is not consistent in cytoplasm and pH_i_ distribution in the cell has been investigated (Shi et al., 2012; Chen et al., 2013). So the pH_e_ is considered to be inconsistent since pH_e_ change is related with the ions and solution exchanges on both sides of cell membrane. The pH_e_ difference and gradient in different position is really anticipated in the future. Thirdly, in our experiments, the pH sensor was placed on the cell membrane, and hence the results cannot accurately describe the activities inside the cell. At present, we are constructing rapid and low invasive injection method of the nanobead sensor into the cytoplasm (Zhong et al., 2016). In the future work, pH_i_ will be investigated after the pH sensor is injected in cytoplasm. Then the comparison between pH_i_ and pH_e_ can improve our understanding of the metabolic pathway of the cell and also the pH gradients on both sides of cell membrane.

## Author contributions

Most of the experimental datas and images have been taken by HL. The experiments of sensor fabrication and sensitivity calibration have been done in Arai Laboratory (in Nagoya) and the virus infection experiment and measurements have been done in Honda Laboratory (in Tokyo) with the assistance of AH, TM, and HM. The paper has been written by HL and revised by FA and HM. All of the authors have contributed to the discussions of the paper writing.

### Conflict of interest statement

The authors declare that the research was conducted in the absence of any commercial or financial relationships that could be construed as a potential conflict of interest.

## Abbreviations

pH_i_: intracellular pH
pH_e_: extracellular pH.

## References

[B1] Allison A. C. (1963). Activation of lysosomal enzymes in virus-infected cells and its prossible relationship to cytopathic effects. *J. Exp*. Med. 117, 879–887. 10.1084/jem.117.6.879 PMC2137597 14012175

[B2] Betakova T. (2007). M2 protein-A proton channel of influenza A virus. *Curr. Pharm*. Des. 13, 3231–3235. 10.2174/138161207782341295 18045172

[B3] Chen S. Hong Y. Liu Y. Liu J. Leung C. W. T. Li M. (2013). Full-range intracellular pH sensing by an aggregation-induced emission-active two-channel ratiometric fluorogen. *J. Am. Chem*. Soc. 135, 4926–4929. 10.1021/ja400337p 23506236

[B4] Ciampor F. Thompson C. A. Grambas S. Hay A. J. (1992). Regulation of pH by the M2 protein of influenza A viruses. Virus Res. 22, 247–258. 10.1016/0168-1702(92)90056-F 1626420

[B5] Gillies R. J. Zaguilan R. M. Peterson E. P. Perona R. (1992). Role of intracellular pH in mammalian cell proliferation. Cell. Physiol. Biochem. 2, 159–179. 10.1159/000154638

[B6] Guinea R. Carrasco L. (1995). Requirement for vacuolar proton-ATPase activity during entry of influenza virus into cells. *J*. Virol. 69, 2306–2312. 10.1128/jvi.69.4.2306-2312.1995 PMC188901 7884876

[B7] Harvey B. J. (1988). Role of Na^+^/H^+^ exchange in the control of intracellular pH and cell membrane conductances in frog skin epithelium. J. Gen. Physiol. 92, 793–810. 10.1085/jgp.92.6.793 3265145 PMC2228922

[B8] Hui E. K. Nayak D. P. (2001). Role of ATP in influenza virus budding. Virol 29, 329–341. 10.1006/viro.2001.1181 11883197

[B9] Liu H. Maruyama H. Masuda T. Honda A. Arai F. (2014). Multi-fluorescent micro-sensor for accurate measurement of pH and temperature variations in micro-environments. Sens. Actuators B. Chem. 203, 54–62. 10.1016/j.snb.2014.06.079

[B10] Liu Y. H. Dam T. H. Pantano P. (2000). A pH-sensitive nanotip array imaging sensor. *Anal. Chim*. Acta 419, 215–225. 10.1016/S0003-2670(00)00988-0

[B11] Maruyama H. Fukuda T. Arai F. (2009). Functional gel-microbead manipulated by optical tweezers for local environment measurement in microchip. Microfluid. Nanofluidics. 6, 383–390. 10.1007/s10404-008-0401-6

[B12] Maruyama H. Masuda T. Arai F. (2013). Selective injection of fluorescence sensor encapsulated in the functional lipid capsule for intracellular measurement, in 26th 2015 International Symposium on Micro-NanoMechatronics and Human Science (Nagoya), 1–2.

[B13] Masuda T. Maruyama H. Honda A. Arai F. (2014). Virus enrichment for single virus infection by using 3D insulator based dielectrophoresis. PLoS ONE 9:e94083. 10.1371/journal.pone.0094083 24918921 PMC4053322

[B14] Moore L. L. Bostick D. A. Garry R. F. (1988). Sindbis virus infection decreases intracellular pH: alkaline medium inhibits processing of sindbis virus polyproteins. Virology 166, 1–9. 10.1016/0042-6822(88)90139-0 2842937

[B15] Oyama K. Takabayashi M. Takei Y. Arai S. Takeoka S. Ishiwata S.. (2012). Walking nanothermometers: spatiotemporal temperature measurement of transported acidic organelles in single living cells. Lab Chip. 12, 1591–1593. 10.1039/c2lc00014h 22437040

[B16] Pinto L. H. Holsinger L. J. Lamb R. A. (1992). Influenza virus M2 protein has ion channel activity. Cell 69, 517–528. 10.1016/0092-8674(92)90452-I 1374685

[B17] Pinto L. H. Lamb R. A. (2006). The M2 proton channels of influenza A and B viruses. J. Biol. Chem. 281, 8997–9000. 10.1074/jbc.R500020200 16407184

[B18] Sardini A. Amey J. S. Weylandt K. H. Nobles M. Valverde M. A. Higgins C. F. (2003). Cell volume regulation and swelling-activated chloride channels. *Biochim. Biophys*. Acta 1618, 153–162. 10.1016/j.bbamem.2003.10.008 14729152

[B19] Shi W. Li X. Ma H. (2012). A tunable ratiometric pH sensor based on carbon nanodots for the quantitative measurement of the intracellular pH of whole cells. Angew. Chem. Int. Ed. Engl. 124, 6538–6541. 10.1002/ange.201202533 22644672

[B20] Singh V. N. Singh M. August J. T. Horecker B. L. (1974). Alterations in glucose metabolism in chick-Embryo cells transformed by rous sarcoma virus: intracellular levels of glycolytic intermediates. Proc. Natl. Acad. Sci. U.S.A. 71, 4129–4132. 10.1073/pnas.71.10.4129 4372608 PMC434342

[B21] Steinhauer D. A. Wharton S. A. Skehel J. J. Wiley D. C. Hay A. J. (1991). Amantadine selection of a mutant influenza virus containing an acid-stable hemagglutinin glycoprotein: evidence for virus-specific regulation of the pH of glycoprotein transport vesicles. Proc. Natl. Acad. Sci. U.S.A. 88, 11525–11529. 10.1073/pnas.88.24.11525 1763066 PMC53168

[B22] Takeda M. Pekosz A. Shuck K. Pinto L. H. Lamb R. A. (2002). Influenza A virus M2 ion channel activity is essential for efficient replication in tissue culture. *J*. Virol. 76, 1391–1399. 10.1128/JVI.76.3.1391-1399.2002 PMC135863 11773413

[B23] Yin L. He C. Huang C. Zhu W. Wang X. Xu Y. (2012). A dual pH and temperature responsive polymeric fluorescent sensor and its imaging application in living cells. Chem. Commun. (Camb). 48, 4486–4488. 10.1039/c2cc30404j 22460168

[B24] Zhong J. Liu H. J. Maruyama H. Masuda T. Arai F. (2016). Continuous-wave laser-assisted injection of single magnetic nanobeads into living cells. Sens. Actuators B. Chem. 230, 298–305. 10.1016/j.snb.2016.01.149

